# Engaging indigenous and academic knowledge on bees in the Amazon: implications for environmental management and transdisciplinary research

**DOI:** 10.1186/s13002-016-0093-z

**Published:** 2016-06-20

**Authors:** Simone Athayde, John Richard Stepp, Wemerson C. Ballester

**Affiliations:** Tropical Conservation and Development Program (TCD), Center for Latin American Studies, University of Florida – UF, 381 Grinter Hall, PO Box 115530, Gainesville, 32611-5530 FL USA; Department of Anthropology, University of Florida, 1112 Turlington Hall, Gainesville, FL 32611-7305 USA; Instituto Federal do Espírito Santo, Núcleo de Estudos em Agroecologia – NEA, Rodovia ES 080, KM 93. São João de Petrópolis, S/N, 29.660-000 Santa Teresa, ES Brazil

**Keywords:** Indigenous and academic knowledge systems, Ethnoecology, Transdisciplinary knowledge production, Brazilian stingless bees, Xingu Indigenous Park, Palavras-chave, Sistemas de conhecimento indígenas e acadêmicos, Etnoecologia, Conhecimento transdisciplinar, Abelhas sem ferrão, Abelha européia africanizada, Parque Indígena do Xingu

## Abstract

**Background:**

This paper contributes to the development of theoretical and methodological approaches that aim to engage indigenous, technical and academic knowledge for environmental management. We present an exploratory analysis of a transdisciplinary project carried out to identify and contrast indigenous and academic perspectives on the relationship between the Africanized honey bee and stingless bee species in the Brazilian Amazon. The project was developed by practitioners and researchers of the Instituto Socioambiental (ISA, a Brazilian NGO), responding to a concern raised by a funding agency, regarding the potential impact of apiculture development by indigenous peoples, on the diversity of stingless bee species in the Xingu Park, southern Brazilian Amazon. Research and educational activities were carried out among four indigenous peoples: Kawaiwete or Kaiabi, Yudja or Juruna, Kīsêdjê or Suyá and Ikpeng or Txicão.

**Methods:**

A constructivist qualitative approach was developed, which included academic literature review, conduction of semi-structured interviews with elders and leaders, community focus groups, field walks and workshops in schools in four villages. Semi-structured interviews and on-line surveys were carried out among academic experts and practitioners.

**Results:**

We found that in both indigenous and scientific perspectives, diversity is a key aspect in keeping exotic and native species in balance and thus avoiding heightened competition and extinction. The Africanized honey bee was compared to the non-indigenous westerners who colonized the Americas, with whom indigenous peoples had to learn to coexist. We identify challenges and opportunities for engagement of indigenous and scientific knowledge for research and management of bee species in the Amazon. A combination of small-scale apiculture and meliponiculture is viewed as an approach that might help to maintain biological and cultural diversity in Amazonian landscapes.

**Conclusion:**

The articulation of knowledge from non-indigenous practitioners and researchers with that of indigenous peoples might inform sustainable management practices that are, at the same time, respectful of indigenous perspectives and intellectual property rights. However, there are ontological, epistemological, political and financial barriers and constraints that need to be addressed in transdisciplinary research projects inter-relating academic, technical and indigenous knowledge systems for environmental management.

## Resumo

**Background:**

Este artigo contribui para o desenvolvimento de abordagens teóricas e metodológicas visando engajar conhecimentos indígenas e acadêmicos para o manejo e a gestão ambiental. Apresenta-se uma análise exploratória de um projeto transdisciplinar desenvolvido para identificar e contrastar perspectivas indígenas e acadêmicas sobre a relação entre as espécies de abelhas sem ferrão e a abelha européia africanizada na Amazônia brasileira. O projeto foi desenvolvido por técnicos e pesquisadores do Instituto Socioambiental (ISA, Ong brasileira) respondendo a uma preocupação levantada por uma agência de financiamento, relacionada com um potencial impacto da produção comercial de mel de abelhas por povos indígenas sobre a diversidade de espécies de abelhas sem ferrão no Parque Indígena do Xingu, no sul da Amazônia brasileira. Atividades educativas e de pesquisa foram realizadas junto aos povos Kawaiwete (conhecido como Kaiabi), Yudja or Juruna, Kīsêdjê or Suyá and Ikpeng or Txicão.

**Métodos:**

Foi aplicada uma abordagem qualitativa construtivista, envolvendo revisão de literatura e realização de entrevistas semi-estruturadas com idosos e líderes, grupos focais, saídas a campo e oficinas nas escolas de quatro aldeias. Aspectos do conhecimento acadêmico e técnico foram levantados através da condução de entrevistas semi-estruturadas e por e-mail, envolvendo especialistas acadêmicos e técnicos.

**Resultados:**

Observou-se que em ambas as perspectivas indígenas, acadêmicas e técnicas, a diversidade é um aspecto fundamental para manter espécies exóticas e nativas em equilíbrio e, assim, evitar concorrência e competição exacerbadas que podem levar à extinção ou desaparecimento de espécies. A abelha europeia africanizada foi comparada com os ocidentais não-indígenas que colonizaram as Américas, e com os quais os povos indígenas tiveram que aprender a conviver. Foram identificados desafios e oportunidades para engajar sistemas de conhecimento indígenas e acadêmicos na pesquisa e manejo de espécies de abelhas na Amazônia. Tanto a apicultura como a meliponicultura, se realizadas de forma artesanal em pequena escala, são vistas como atividades com potencial de contribuir para manter a diversidade biológica e cultural em paisagens amazônicas.

**Conclusão:**

A articulação de conhecimentos de acadêmicos e técnicos não-indígenas com sistemas de conhecimento indígena pode informar práticas de gestão sustentáveis que reconheçam e respeitem as perspectivas e direitos de propriedade intelectual indígenas. Entretando, existem barreiras ontológicas, epistemológicas, políticas e financeiras que devem ser enfrentadas em iniciativas de produção transdisciplinar de conhecimento inter-relacionando diferentes sistemas de conhecimento.

## Background

In academia, there has been much discussion on the value of articulating indigenous and scientific or academic knowledge to conserve biodiversity and promote sustainable environmental management [1–6]. Hall ([7]:328) considers traditional ecological knowledge (TEK) as a “*component of social capital for promoting economic progress and supplying environmental services*” which has been neglected by official planners and policy-makers. Cultural understandings of the environment might be instrumental in nature conservation initiatives and programs, providing knowledge of species requirements, ecosystem dynamics, sustainable harvesting levels and ecological interactions [8–10]. Baggethun et al. [11] highlight the importance of traditional ecological knowledge systems (TEK) as reservoirs of experiential knowledge that might provide insights for the design of adaptation and mitigation strategies to cope with global environmental change.

Berkes et al. [12] define traditional or indigenous knowledge (IK) as cumulative and adaptive by nature, tested by trial-and-error and transmitted through generations orally or by shared practical experiences. According to Brush [1], TEK reflects the ecological adaptation of humans to diverse environmental settings, thus it can serve as a ground for the development of initiatives to conserve biological diversity. Erren et al. [13] propose that folk knowledge held by non-scientists such as indigenous persons may be manifested in common sense. These bodies of knowledge might be compared to the scientific research process, since they are the result of powerful tests of hypotheses by many individuals across time and space. Evidence supports the claim that biodiversity conservation and management projects have been more successful when local knowledge was incorporated in the process [9, 14, 15].

Despite existing examples integrating indigenous and academic “bodies of knowledge” towards solving complex problems, the processes through which such knowledge engagement might be enabled or constrained are poorly elicited, as Raymond et al. [6] noted. Engaging indigenous and academic knowledges for solving environmental problems may be challenging and time consuming. It entails skills and tools for transdisciplinary knowledge production, long-term funding and coordinated actions by different actors including indigenous communities, policy-makers, researchers, government officers, managers and others [16, 17]. In academia, generating knowledge to address complex social-environmental problems often involves developing interdisciplinary research across disciplinary fields in the biophysical and social sciences [18]. Transdisciplinary research involves co-production of knowledge by scientists, researchers and non-academic participants [17, 19]. Both interdisciplinary and transdisciplinary knowledge production face epistemological, methodological, philosophical, political, financial and practical obstacles [6, 16, 20, 21].

Given the multiplicity of contexts and objectives that underlie co-production of knowledge between non-academic and academic actors, we suggest that such initiatives should provide analyses and reflection on both the process and the products or outcomes achieved, enabling adaptive learning and knowledge exchange across geographical and cultural borders.

In this paper, we present an analysis of a project developed to engage indigenous and academic knowledge on the ecology and management of bees in the Amazon. The project was implemented in response to a concern from a funding agency, related to the potential impact of development of apiculture on the diversity of stingless bees in the Xingu Park region. We adapt the framework proposed by Raymond et al. [6] to evaluate how knowledges were identified, engaged, evaluated, integrated and applied within an educational and research project named “Bees Ecology”, involving four indigenous peoples from Xingu Park. Project activities included consultation with biophysical scientists and practitioners specialized in bee biology, ecology and management in Brazil. We reflect on “how” knowledges were engaged, present “what” knowledge resulted from this effort, analyze “whom” the produced knowledge is useful to and for “what” purpose. We add reflections and questions to the framework, which may be useful to inform transdisciplinary research between scientists, practitioners, managers and indigenous communities involved in initiatives of collaborative knowledge production.

The article is developed in five parts: a) background on the context and framing the problem, including two introductory subsections on historical and biological elements of the Africanized honey bee and stingless bee species; b) methods and tools used for transdisciplinary knowledge assessment and engagement; c) indigenous knowledge elements on stingless bees including myths and ethnoecology; d) academic, technical and indigenous perspectives on the relationship between the Africanized honey bee and stingless bee species in the Brazilian Amazon; and e) contributions to collaborative environmental management engaging indigenous and academic knowledge systems.

### Brief history of the introduction of the Africanized honey bee in Brazil

According to the official story written by non-indigenous scholars, the introduction of the European *Apis mellifera* in Brazil occurred around 1839 by the Portuguese, mainly by the Jesuits priests, primarily for wax extraction for candle production used for religious purposes [22]. In 1845, German colonizers brought over more bees, beginning apiculture in the south of Brazil [22]. Following these events, other colonizers also brought European bees to different Brazilian regions, some of them unregistered. Until the middle of 20^th^ Century, the European *A. mellifera* did not disperse beyond the locations where it was introduced.

The Brazilian government asked Dr. Warwick Kerr, a biologist and geneticist, to “create” a bee that could produce more honey in tropical environments. In 1956, Dr. Warwick Kerr led an expedition to South Africa and Zimbabwe and brought 36 African queens to an agricultural research station in the State of São Paulo. By interbreeding the queens through artificial insemination with European drones, Kerr and his associates produced a number of first generation hybrids. After several months, their stock of Africanized honey bees was reduced to 29 and they were maintained in hive boxes equipped with queen excluders. In October of 1957, a local beekeeper noticed the queen excluders and removed them, accidentally releasing 26 Africanized honey bee queens with small swarms to the forest nearby [22, 23]. There was no way to find these “lost queens” again. This incident changed the history of bees and beekeepers forever. Africanized honey bees have spread out to the west and north in South America, Central America and eastern Mexico, at a rate of near 200 miles per year. In 1990 these bees reached southern Texas, finding their way to California in 1995. They then spread north, and were found in Nevada by 1998. By 2004, the bees had migrated through Texas and were detected in Oklahoma. Most recently, the Africanized honey bee has become established in western Louisiana, southwestern Arkansas, and southern Florida [24].

### Biological and ecological traits of the Africanized honey bee and stingless bees

Since the introduction and hybridization of the Africanized honey bee in Brazil some sixty years ago, there has been a lot of debate and research on the possible impact of this exotic bee on the diversity of stingless native bees and other pollinators, on the ecology of tropical forests, and on the pollination of economically important crops [25–31].

Genetically speaking, the Africanized honey bee is a hybrid of one of the several European honey bee subspecies (*Apis mellifera*; *A. m. carnica*; *A. m. caucasia*; or *A. m. linguica*) and the African honey bee (*A. m. scutellata*) [25]. The Africanized honey bee is classified as an r-selected species: they discover new habitats quickly, disperse readily to find other habitats when their current one has become unstable or inhabitable, use resources efficiently and reproduce rapidly. Furthermore, they have a highly defensive nature and show the ability to survive on sparse supplies of pollen and nectar [25, 32]. According to Roubik [33], stingless bees have 50 times more species that the genus *Apis*, and biologically differ from *Apis* species in many aspects. First, stingless bees cannot migrate, and are restricted to local and regional ecological and climatic conditions. Stingless bees produce less honey when compared to Africanized honey bees, and thus they possess less economic importance. Nesting habits are greatly variable among stingless bees. They generally lack the generalized nesting habits of the Africanized honey bee, and the capacity to abscond as colonies. While there is evidence of competition for nest sites and food between *Apis* honey bees and meliponine stingless bees, Roubik (op cit.) affirms that competition is comparatively more intense among honey bees, than between these and stingless bees.

Stingless bees are also named meliponines, since most of them belong to the Meliponini tribe in the Apidae family. They play a fundamental role in the maintenance of biodiversity in the tropics, and are responsible for the pollination of some 80 % of tropical plant species [34]. Brazilian ecosystems host around 5,000 species of native bees, which is nearly 20 % of the world’s bee diversity [35]. Apiculture and meliponiculture (keeping of stingless bees) are promising market-oriented economic alternatives for indigenous peoples and other traditional societies. Besides the economic benefits from increasing market opportunities for bee products, meliponiculture can generate important information to be used in the conservation of native bee species around the world [34, 36, 37].

While there has been significant research done on the biology, ecology, beekeeping and conservation of Brazilian stingless bees [37–42], there is a gap of knowledge regarding indigenous knowledge of stingless bees species and indigenous peoples views on the impact of the Africanized honey bee over stingless bees diversity. Darrell Posey [42–46] carried out important research on Kayapo ethnoentomology, including Kayapo knowledge and management practices of stingless bees. He pointed out that indigenous knowledge on the relationship of Africanized Honey bee and the native stingless bees is an issue that deserves more attention and further study, and that research on IK represents a central issue in the conservation of both cultural and biological diversity in Amazonia [42, 43].

In the past several years, the world has witnessed an unprecedented collapse of Africanized honey bee colonies, a new phenomenon known as Colony Collapse Disorder (CCD), which has been especially severe in the US. This phenomenon is caused by a complex combination of factors such as pathogens, bacterial diseases, cumulative impacts of pesticide use and decline in genetic diversity [47]. According to the United States Department of Agriculture (USDA), an estimated one-third of all food and beverages are made possible by pollination, done mainly by honey bees. In the US, pollination contributes to crop production which is worth some $20–30 billion in agricultural production annually [47]. A decline in managed bee colonies puts great pressure on the sectors of agriculture reliant on commercial pollination services.

## Methods

### Study site and context

**Fig. 1:**
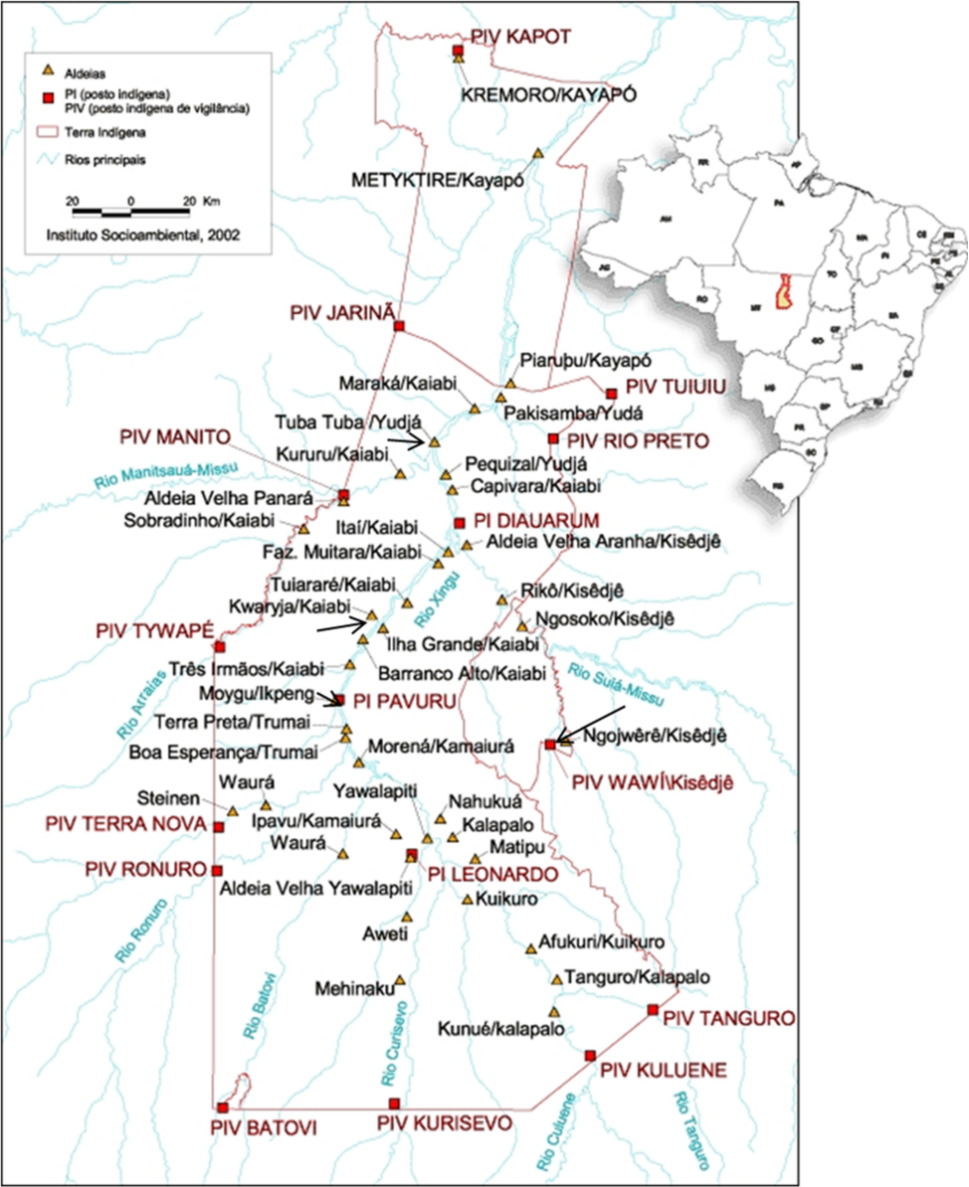
Map of Xingu Indigenous Park, located in Mato Grosso state, Brazil, locating the four villages participating in the Bees Ecology project

The Xingu Indigenous Park was created in 1961 by the Brazilian government. It has an area of 2,642,003 ha within the Xingu River watershed, in a transitional zone between the savannas and the Amazonian tropical forest (Fig. 1). Fourteen indigenous groups live within the Park’s limits, totaling 4,829 people in 2011 [48]. The vegetation of Xingu Park is composed of a mosaic of various ecozones such as savannas, flooded forests, non-flooded forests, palm groupings and forests on growing on “black earth” or anthropogenic soils [49].

The apiculture activity began in Xingu Park in 1996 through the Fundação Mata Virgem, incorporated by the Brazilian NGO Instituto Socioambiental^1^ in 1997 (ISA). Initially, few beehive boxes were installed in bigger villages. In each village, men interested in working as beekeepers began to receive specialized technical training through collaboration with practitioners and technicians from APACAME,^2^ an association of beekeepers from São Paulo State (Associação Paulista de Apicultores Criadores de Abelhas Melíficas Europeias). Gradually, the number of hives increased, as well as the interest in the activity. Up to now, there has been no report of serious accidents or injuries caused by the Africanized honey bee in the Park’s region. Since the beginning, the philosophy of the project was to build and strengthen local community capacity to run the honey production and commercialization, with gradual transfer of financial resources from the NGO to communities through the local organization ATIX (Xingu Indigenous Land Association). Community representatives and ATIX officers have participated in the development of Xingu honey trademark and in other activities related to the organization of honey production and commercialization [50].

In 2001, Xingu honey trademarked as “Mel dos Índios do Xingu” (Xingu Indigenous Honey) was certified as an organic product by the Instituto Biodinâmico (IBD), becoming the first indigenous product to be labelled as organic in Brazil, and receiving the seal from the Brazilian Ministry of Agriculture (SIF). In 2015, apiculture was the most successful market activity developed in Xingu Park, involving around 24 villages and 5 indigenous groups. In 2011, around 900 kg of Xingu indigenous honey was sold to Pão de Açúcar, a well-known Brazilian supermarket chain [50].

Concurrently with the apiculture activity, ISA started to support the development of meliponiculture (stingless bee stewardship) in 1998, nowadays practiced in five villages. The main species being managed are jataí (*Tetragonisca angustula*), tiúba (*Melipona compressipes*), and marmelada (*Frieseomelitta* spp). Most of the stingless bees’ honey production is consumed inside the Park for either medicinal or nutritional purposes.

The problem that informed this initiative was brought by a project evaluator working for the Ministry of the Environment, which managed funds provided by the Fundo de Pequenos Projetos do GEF/PPP/Global Environmental Facility Small Projects Fund. This entity supported the production of *Apis* honey as a sustainable market activity for indigenous groups in the Xingu Park. The project evaluator was concerned about a possible negative impact of commercial beekeeping on the diversity and resilience of stingless bee species in the Park’s region. To address this concern, a project was developed in order to identify, synthesize and engage academic knowledge and indigenous perspectives on the relationship between the Africanized honey bee and stingless bee species in the Park. The “Bees Ecology” project involved participatory research and transdisciplinary knowledge production among four sociolinguistic groups in the Xingu Park, aiming to address the question brought by the funding agency, to synthesize and revitalize local knowledge, and also to inform the development of apiculture and meliponiculture in Xingu Park.

### The Kawaiwete or Kaiabi indigenous people

The Kawaiwete, recently self-designating “Kawaiwete”, speak a language in the Tupi-Guarani subfamily and originally occupied a large territory in the Tapajos river watershed [51]. They have vigorously resisted the invasion of their lands by rubber tappers since the end of the 19th Century. After the 1950s, the region crossing the Arinos, Peixes and Teles Pires rivers was divided up into lots that became ranches and the Kawaiwete were divided into three groups. Most were transferred to the Xingu Indigenous Park between 1950 and 1966. Currently, the Kawaiwete from Xingu are distributed in nine villages in the northern portion of Xingu Park, totaling nearly 1.000 people. They play an important role in Xingu Park’s ethnopolitics through ATIX, a local multiethnic indigenous organization created in 1995 [52].

### The Yudja or Juruna indigenous people

The Yudja (self-designation, commonly known as Juruna) are a canoe people, speaking an isolated language in the Tupi-Guarani subfamily, who have long inhabited the islands and peninsulas of the lower and middle Xingu, in Pará state [53]. In the beginning of the 20th century, they migrated upriver, running away from rubber tapers, settlers and Kayapo indigenous groups. Today, they total nearly 378 people divided into four villages [53]. The Yudja created their own Association, named Yarikayu, in 2002.

### The Kīsêdjê or Suyá indigenous people

According to Seeger [54], the Suyá, or Kīsêdjê (self-designation) are the only group of the Gê linguistic family in the Xingu Indigenous Park. Since their arrival in the region (probably in the second half of the 19th century), they have adopted many new habits and technologies triggered by the contact with other Xingu groups and, primarily, with those of the so-called “cultural area of the Upper Xingu”. Despite these cultural exchanges, they never abandoned their cultural singularity. In 2002 the Kĩsêdjê moved from their old village (Rikô) located in the Suyá River, to their new village Ngojwêrê, placed in a sacred region at the Wawi Indigenous Land, adjacent to the Xingu Park in its middle-east portion. Currently, they are distributed in three main villages within Wawi land, totaling near 330 people in 2010 [55].

### The Ikpeng or Txicão indigenous people

The Ikpeng (commonly known as “Txicão”) are a Carib-speaking group that came from the region of the feeder streams of the Xingu in the beginning of the 20^th^ Century, when they lived in a state of war with the upper Xinguan neighbors. Contact with the non-indigenous society was even more recent, at the beginning of the 1960s, and had disastrous consequences for their population, which was reduced to less than half as a result of diseases and killings. They were then transferred to the borders of the Xingu Indigenous Park, totaling around 450 people in 2012 [56]. Most of the population live at the Moygu village, adjacent to the Pavuru Indigenous Post in the middle region of Xingu Park. They have indigenous schools, indigenous filmmakers and maintain alliances with other indigenous groups in the Park, but nevertheless their society is quite distinct [56].

### Methods

A qualitative methodological approach was developed for the Bees Ecology project, including participant observation, field walks, mapping activities, focus groups and participatory workshops in four villages. School and field activities involved elders, shamans, beekeepers, indigenous teachers, women, and school children.

Information on scientific names for bee species presented in this article are based on published literature. Since the project was developed through the indigenous schools in each village, indigenous teachers authorized the publicizing of the project’s results in reports and in this article. Participants preferred to be identified by their real names.

**Fig. 2:**
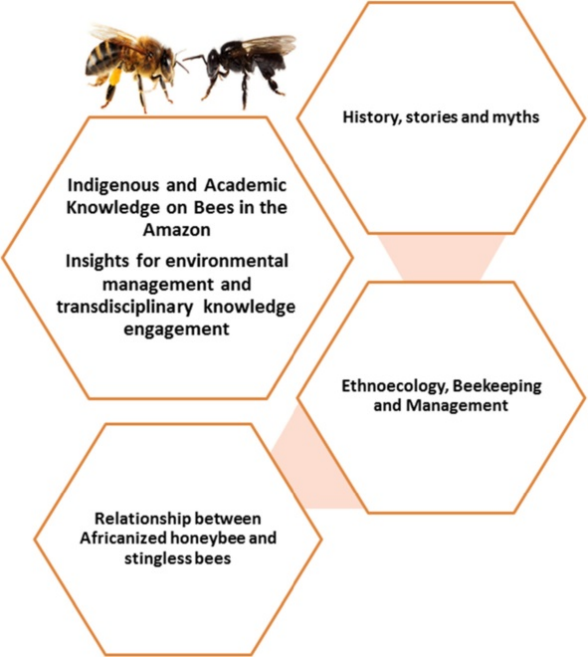
Main themes approached in the Bees Ecology project in the Xingu Indigenous Park, Brazilian Amazon

Literature review on academic knowledge and field and school activities in the villages were developed concomitantly. Indigenous perspectives and knowledge on history, ecology and management practices for different stingless bee species and the introduced honey bee were synthesized, and then compared and inter-related with non-indigenous academic and technical knowledge. The research question or problem was defined by the funding agency, and was used to guide the different activities during the project development. The main themes approached in the Bees Ecology project are summarized in Fig. 2. The problem-centered research question was: to what extent the production and marketing of Africanized honey bee honey by indigenous communities in Xingu Park might threaten the sustainability of stingless bee species in the region?

We interviewed elders and shamans to get their perspectives on the four main themes approached by the project, followed by organization of school workshops, and by field visits for preliminary mapping of ecological zones and to discuss elements on bee ecology, taxonomy and management with indigenous students and community representatives on-site. After each activity, we would meet in the school for synthesizing the information and data gathered. Class exercises and individual and group interviewing were carried out to collect and summarize information on competition for pollen, nectar or nest niche between the honey bee and the stingless bee species, and among stingless bee species. We created scenarios, where one species was compared to others in terms of aggressive behavior, and tendency to fight for pollen, nectar or nesting resources.

In order to compare indigenous perspectives and viewpoints with those from non-indigenous technicians and academics, semi-structured interviews were conducted with four “experts” from different institutions and with various levels of expertise in beekeeping and bee ecology. The results of these interviews were compared with indigenous peoples’ opinions, as well as with information from bibliographic sources. Personal and remote interviews (by e-mail) were conducted with Dr. Paulo Nogueira Neto (University of São Paulo, retired); APACAME representatives Dr. Constantino Zara Filho, Waldemar Ribas Monteiro and Mário Isao Otsuka; Dr. Vera Lúcia Imperatriz-Fonseca (University of São Paulo, USP), Dr. Astrid Kleinert (University of São Paulo, USP) and Fernando Oliveira (Beekeeper, Iraquara Project). These experts were chosen because they represent different disciplinary and technical backgrounds related to the biology and ecology of native and introduced bees, and to technical and practical issues of bee domestication and management. For assessing and reflecting on the process of knowledge engagement, we adapted the framework developed by Raymond et al. [6].

## Results and discussion

The project resulted in products tailored to indigenous communities, a report presented to the funding agency, and academic publications [57, 58]. It also resulted in the organization of a special session during the 13^th^ Congress of the International Society of Ethnobiology, in which Kawaiwete and Kayapo myths were developed in an interactive storytelling format using puppets [59]. In addition to addressing the problem through a transdisciplinary approach, the project enabled, with limitations, the sharing of knowledge within and between the indigenous groups involved in apiculture and meliponiculture activities, as well as between them and academic “experts” in Brazil, within the leading NGO (ISA), and with funding agency personnel.

**Fig. 3:**
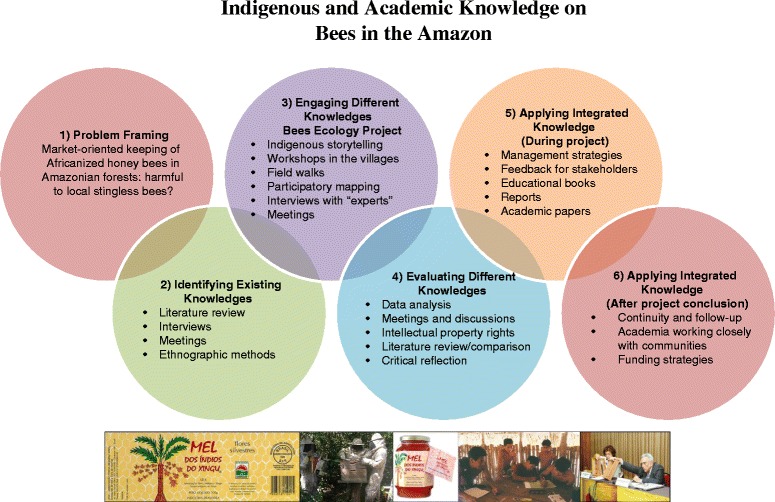
Engaging indigenous and scientific knowledge on bees in Xingu Park, based on the framework by Raymond et al. [6]. Photo documentation strip shows the label and the "Mel dos Índios do Xingu" (Honey from the Xingu Indians) product from keeping of Africanized honey bees in Xingu Park; indigenous beekeepers at work; meetings, workshops and activities in schools; and academic experts Dr. Vera Lucia Imperatriz-Fonseca and Dr. Paulo Nogueira Neto, who participated in the Bees Ecology project. Photos by Simone Athayde and Wemerson Ballester, photo of academic experts used under permission from Dr. Vera Lucia Imperatriz-Fonseca

The results and discussion are presented in the order in which the themes were developed with the indigenous communities involved in the project, adapting the framework by Raymond et al. [6] as an analytical lens to reflect on the process of knowledge identification, engagement, evaluation and application (Fig. 3):

- Oral history and myths related to the Africanized honey bee and stingless bees;
- Elements of ecology and management of bee species by indigenous communities in Xingu Park;
- Academic and indigenous perspectives on competition between bees and on the relationship between the Africanized honey bee and stingless bee species;
- Reflection on the process of knowledge engagement and recommendations for knowledge exchange, research and management of bee species between academics, practitioners and indigenous communities.

### Between history and myth: the encounter of indigenous peoples with the Africanized honey bee

The educational project “Bees Ecology” was initiated with a module on “History and Stories”, involving students and community representatives, asking them to tell their stories about the Africanized honey bee and the stingless bee species. We then told the academic or “official story” of the arrival of the Africanized honey bee in Brazil, summarized in the introductory section of this article.

After the breeding between subspecies of *Apis mellifera* happened in 1956, this introduced hybrid spread quickly to other Brazilian regions. Indigenous communities in Xingu Park told stories portraying their first encounter with the “new” bee, saying that they were surprised to find a stinging bee that could produce honey, thinking that it could be a kind of wasp. The Yudja and the Kawaiwete named the honey bee as “honey wasp”. Representatives of the four groups involved in this research said that in the first contacts with this new bee, they did not let the children eat its honey, because they thought it could make the kids sick or induce vomit.

The Kawaiwete were already informed about the existence of the honey bee, because when the Park was created, around 1961, the Villas Bôas brothers had warned them about this new bee species brought to Brazil. The Yudja affirmed that when they first met the honey bee, it was not very aggressive, and they could extract its honey without using fire. Later, it began to behave more aggressively, and they thus started to use fire.

According to the Kawaiwete, the first meeting with the introduced honey bee was a bit traumatic. The elder Masi’a from Tuiararé village mentioned that he found this bee around 1964, thinking that is was another type of stingless bee. But then, the bees started to sting him and he had to run to the forest, asking his wife to run also. He had fever because of the stings. Then, later on, he met his friend Tymaka’i, and both collected the honey using fire.

The Kīsêdjê first thought that it was a species that they already knew, but later on they named it differently. Representatives of the Ikpeng people mentioned that for them, the introduced honey bee was a native bee just like the other stingless bees, differing mainly because of its stingy capacity. One elderly woman commented that it was the first time that somebody told them the honey bee story, and that she did not not believe that the Africanized honey bee was brought from outside Brazil.

Tximairu Ikpeng, an elderly woman who participated in the encounter with the exotic bee, told us that the first Ikpeng to encounter the Africanized honey bee was Papru, a great shaman. He was coming back to the village after a visit to the swidden garden to harvest cassava, along with his wives. Arepó, one of his wives, saw a bee up in a hole of a tree and called the others: − Come on, here is the “ae” bee. She thought it was another bee species. Papru came to cut down the tree and saw that it was another type of bee, which he named “teregyum” (blue eyes bee). In the beginning, only Papru could eat this honey, and the people in the village, when encountering this bee, used to bring honey for Papru. After people discovered that the honey was good and did not do any harm, everybody began to eat it, and they did not bring honey to Papru again.

Kawaiwete elder Tujarajup told us that they believe that all natural resources have spirits, and only the shamans have the power to communicate with these supernatural beings and cure illnesses provoked by them. As Posey [43] noted, myth is an important vehicle for transmission of ecological knowledge. In Kayapo cosmology, an ancient shaman called “wayanga” taught their ancestors how to live, work and defend themselves like social insects, gaining his knowledge observing bee, wasp and ant behavior [45, 60]. Traditional circular villages are said by the Kayapo to take the cross-sectional form of conical nests of wasps and bees. Studying Kayapo’s knowledge on bees and insects, Darrell Posey [42] observed that bee specialists among the Kayapo from Gorotire were all shamans.

In the Kawaiwete cosmology, the bees have their own Master or Spirit, who takes care of them. This spirit regulates both the reproduction of the bees and the production of honey. Each indigenous group has its own myths and beliefs related to the bees, but interestingly enough, there are some common features across different indigenous cosmologies. First, there are the relationships between bees and heaven, and sometimes with thunder. The Ikpeng sing a song given by a bee to avoid thunders during storms. They say that this song is very dangerous and should not be sung when there are no storms. According to Posey [45], in Kayapo of Gorotire cosmology, a powerful shaman named *Bepkôrôrôti* was taken into the sky in a flash of lightning, residing in the clouds and having the power to send lightning, thunder and rain. To show respect and reverence for *Bepkôrôrôti*, who is a great honey consumer, the Kayapo leave a portion of the brood comb and honey for him every time they gather honey. For the Kayapo, honey, wax and bees are associated with the heavens and rains, and beeswax is burned to produce a smoke that is believed to attract storm clouds and rains. The Kayapo believe that this smoke “…*repels evil spirits, purge houses from lingering ancestral spirits and protect children from witchcraft*” [39: 135]. The Pälawan of Indonesia burn the wax of a small stingless bee called “*kätih*” to stop thunderstorms [61]. In Yudja cosmology, the bees came from heaven and their Master is “Selã’ã”, a divinity who created all living beings. He sent the bees to the forest, so people could eat their honey. The bees use the rain to make the honey cold, tasteful and flower smelling.

Ntoni Suyá, shaman at Ngojwêre village told us that for them, the bees sing and have festivals. The spirits, in the feasts, dance and use the honeycombs as earrings, in the same way that the Kĩsêdjê used to wear round earrings. They say that this adornment is very important for the spirit of the bee. The feasts only happen in the hives of bees that live in big holes in the trees. All the bees are invited for the festivals, but there is one bee, called Kangárá (commonly known in Brazil as “caga-fogo”, *Oxytrigona* sp.), who cannot enter, because she is too “hot” (referring to its aggressive behavior and to the burning power of its sting). So, she stays outside, dancing in the entrance. Only the shaman can talk to the spirit of the bees, who teaches him music from the bees. Later on, the shaman sings the music for the people in the village, so that they can also learn. Through this way, the Kĩsêdjê always learn new music. There are birds who eat bees or who take care of the bees, such as the “xapi” for the Yudja and the “kakê” for the Kĩsêdjê (unknown species). For the Kĩsêdjê, the firefly is a bee spirit, who flies at night. They believe that if you fasten a cotton thread around its neck and hang it in your hammock, it will show the direction of bee nests with its head, when asked.

**Fig. 4:**
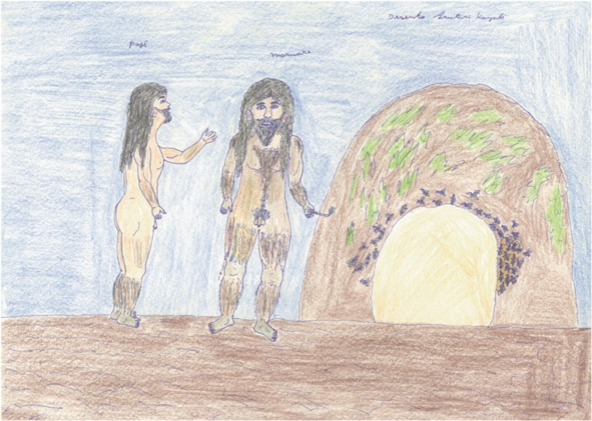
Maruari, the spirit of the Africanized honey bee. Drawing by Arutari Kaiabi (male, 12 years old), Kwaruja village, Xingu Park

In Kawaiwete mythology, bees are related to other animals that sometimes created them and other times only take care of them. According to Tujarajup, shaman and chief of the Kwaruja village, Kawaiwete people believe that there is a spirit who is the father of the honey, named “eirup” (eit = honey, tup = father). The spirit that takes care of the native bees is called “Tajuipa”. The Africanized honey bee also has her own spirit, called “Maruari”. When he is happy and sings bee songs, there is a great number of honey bees in the village. Tuiarajup affirmed that is Maruari who controls honey production, having also influence in the apiculture activity (Fig. 4).

The following text is based on a story told by Tujajarup, written by Sirawan Kaiabi, teacher of Kwaruja village, and translated here:

“*Maruari is the spirit owner of the honey bee, he commands everything that happens with this bee. He does not like that people burn bee wax, and nobody can kill the bee without reason. When the spirit sings the music of the bees, they spread all over the places. That’s why sometimes there are a lot of bees.*

*Only the shaman knows this spirit, normal people cannot see or talk to him. The visitors cannot enter in his house, only who is fast enough can enter, because there are two bee colonies taking care of the two doors. This spirit is tall, hairy has a beard and long hair. His thighs are perforated, and when there is a lot of pus coming out of his thighs, it is a signal that the bees are going to produce a lot of honey. When his thighs are dry, there is no honey. That’s why sometimes the bees do not produce honey in the box hives. If the owner (spirit) decides that the bees are not going to produce honey, they do not reproduce. Then, some months later you find only larvae, there is no honey. For us indigenous, it is the same, but for the white people it is different.*

*Maruari asks the shaman why the “toryp” (white people) is interfering in his work. The shaman replies: − Because it is being difficult for us, we have to go far to find honey, so the toryp brought the apiculture for us. Maruari says: − The toryp has to guarantee this work for you, so you won’t have problems later. When the beekeepers capture a colony and the bees go away, this happens because the spirit takes them out of the box hive, it is the spirit who takes them away*.”

There are many taboos in regards to honey consumption and honey gathering. The Ikpeng say that some bees are very dangerous and that their spirits keep strange things such as dead bugs, rat skulls, snake skulls, monkey skulls etc. Especially when a couple has a small baby or little kids, they can’t consume honey, because it can cause sickness and even death to the child. Once, Manaku Yudja, beekeeper at Tuba village, commented that his son was very sick. He said that the shaman saw a lot of bee larvae on the boy’s face. He said that this happened because he was practicing apiculture and he couldn’t at that moment; he could do it only after his son grew up. Some stingless bee honey can only be consumed by elderly and shamans. Melobo Ikpeng, chief of Moygu village, asked us to remove the Yiktxi (jataí, *Tetragonisca angustula*) from the village’s beekeeping place because only old men could consume its honey. Meyer-Rochow [62] explains that food taboos among ethnic groups might have different social-ecological histories and purposes. Children and women, especially pregnant, are more often subjected to food taboos in comparison to men. While not all people in the villages were aware of the fact that the Africanized honey bee was an exotic species introduced and hybridized in Brazil by scientists, Kawaiwete shamans mentioned the name of the spirit Master of the introduced bee, and incorporated the new species in their mythology and belief systems in a fairly short period of time.

### Elements of ethnoecology, taxonomy and management of bees in Xingu Park

During the workshops carried out in the schools involving elders, teachers and students, the Kawaiwete and the Yudja identified 42 ethnospecies of stingless bees, the Kīsêdjê identified 35 ethnospecies and the Ikpeng recognized 41 ethnospecies. We define ethnospecies as local species entities for types of bees recognized and named by indigenous persons who participated in this study. As Otieno et al. [63], pointed out, these ethnospecies can match, under-differentiate or over-differentiate as compared to scientific species. Through preliminary participatory mapping exercises carried out in each village, there were identified main ecological zones for the region surrounding each village. Field walks were conducted to each ecological zone to observe and complete the list of ethnospecies developed during school workshops. In his initial research, Posey [60, 64] recorded 56 folk bee species recognized by the Kayapo.

Honey gathering is predominantly a male activity. This agrees with Australian Aboriginal menfolk, who search and collect the honey of native stingless Australian honey bees [65]. Nevertheless, many times the entire family goes in expeditions to collect honey and other forest products. At night, people can find bee colonies by the sound that they produce. Men usually cut down the tree to collect the honey, pollen and larvae, but sometimes they construct ladders in order to access bee nests.

Indigenous taxonomy of bee species can vary from people to people, but there are some common features across their systems of classification, such as the structure of nests entrance and the smell of the bee. The entrance of nests is the main feature used to recognize and sometimes to name a meliponine species. Posey and Camargo [46, 60] also noted this as the main feature used by the Kayapo Mektutyre to classify meliponine species. Other characteristics used in Kayapo bee classification are shape, size, composition, color and smell. According to the shape and composition of nest entrance structures, a bee species can be named after an animal species or parts of the human body, etc. The Yudja people commonly name bees after animals, accompanied by the suffix “wïla”, which means “honey” and awïla means bee. Thus, there is the “takurare wïla”, turtle honey; “tuwã wïla”, tapir honey; “awïla kapa hahayã”, wasp honey (*Apis mellifera*), and so on.

According to the beekeepers participants of this research, the honey bee is classified with wasps rather than with stingless bees, concurring to what Posey [45] recorded for the Kayapo. The Kawaiwete use a mixture of elements to name bee species, and also group some morphologically similar species, differentiating them by the size (small/big) or characteristic/behavior (*tata eit* – fire bee). Similar to Yudja taxonomy, the Kaiwaiwete give animal names to some species, using the suffix “eit”, which means “honey”: for example, “akyky eit”, howler-monkey honey, “mijui’eit”, swallow honey etc. [66].

Bees have been used by indigenous societies since immemorial times for food, medicine, artifact production and, more recently, for market-oriented economic activities. In general, indigenous peoples in Xingu use the pollen, honey, honeycomb and the larvae produced by the Africanized honey bee and by stingless bee species as food products. However, many stingless bee species products are not consumed because of the nasty taste of their honey or due to religious taboos. Honey is also an important energetic source used during forest expeditions and travels. When the Kawaiwete people moved from their ancestral land in Tapajos river watershed to the Xingu region in the 1950’s and 1960’s, they relied mainly on honey, fruits and game during their journey. Villas-Boas [66], presents a comprehensive table of stingless bee species known by the Kawaiwete, accompanied by their indigenous names, uses and scientific designation, if known.

Kawaiwete beekeepers participating in the project activities mentioned that the higher diversity of stingless bee species is found in the non-flooded high forests (mata alta), followed by the forests on black earth soils (capoeiras), flooded forests (mata de várzea), savannas (campos e cerrados) and swidden gardens (roças). According to them, the Africanized honey bee can live in any ecosystem.

A great diversity of melliferous plants can be observed at Xingu Park, distributed across a mosaic of different ecological zones. There is an increased availability of flowers during the dry season, between June and September. According to information collected during interviews and workshops, it is in the non-flooded forests that the majority of melliferous plants and trees for nest building occur. Periodically flooded forests and old fallows also concentrate a significant amount of plants of importance for the stingless bees.

An important aspect linking the diversity of bees with landscape patchiness refers to the role of indigenous societies in creating, enhancing and maintaining biodiversity at local and landscape scales, through biocultural co-evolution. Evidence from previous research has shown that indigenous management practices linked to rotation of settlements and swidden-fallow agriculture have enhanced the patchiness and diversity of vegetation types, plant species and niches for animal species, including bees, in the Amazon [67–72]. Indigenous management practices, intertwined with their belief systems and worldviews, have enhanced the diversity of ecozones and habitats for bee species in the Amazon. We highlight the important role of forest-based agriculture in this process, and the importance of old fallow patches (also known as “capoeiras”) in providing a variety of food resources for bees and other animals.

### Indigenous and academic perspectives on relationships between the Africanized honey bee and stingless bees in Xingu Park

The introduction of the European bee in the American continent, and its subsequent hybridization with the Africanized *honey bee* has received much attention in both academic circles and in governmental environmental and agriculture agencies. The process of adaptation of stingless bees to the newcomer Africanized honey bee was an important theme discussed by academics, practitioners and indigenous persons during the Bees Ecology project. Dr. Nogueira Neto commented that the introduction of the European bee was inevitable, because Brazil needed honey and beeswax, and at that time, techniques for keeping native bees were non-existent.

Chief Kuiussi Kīsêdjê, of Ngojwêrê village, thinks that right after the arrival of *Apis mellifera*, the other bees probably found its presence odd, but with time they got used to it. He never observed fierce fights between the exotic and the local bees. Moreover, there are some bees that can attack and kill the *A. mellifera* too. He affirms that there are flowers for everybody and he sees no problem with the apiculture activity, if the beekeepers are not increasing significantly the number of bees, only taking the families from the wild and putting them in the beehives. He likes the apiculture, because there is more honey for consumption by the community and also for sale.

Arupajup Kawaiwete, a knowledgeable older man from Kwaruja village, compared the arrival of the *A. mellifera* in Xingu to the arrival of a new people in a territory. In the beginning, it is different, but then people begin to talk, fight and know each other until they adapt to the new situation. He says:

“*In the past, this “kaweit” (Apis) did not exist, then it appeared. Like us, other people, you white, we indigenous, other tribes come to us and we find it odd if we do not know the people. We say: “- Who is this?” Maybe for the bee is like this, when the “kaweit” arrived, the little bees found it strange, but… nobody know if the bees speak, maybe they speak. Maybe when “kaweit” arrived, she came and said “hello” to the others, explaining from where she comes from and why she was there. That’s the reason why they are living together, and do not bother one another. In the beginning they liked each other but now with the “europa” spreading all over maybe the native bees are saying: “Wow, they came to take the food we have!*” *Arupajup Kaiabi, Kwaruja village*

Fernando Oliveira, beekeeper and practitioner, who works in the Iraquara Project in Amazon State, has not observed a negative impact of *the Africanized honey bee* in forest ecosystems, adding that the exotic bee does not adapt well in forests with low luminosity and high humidity such as the Amazonian forests, preferring hot and sunny environments.

Scientists have examined the impacts of the Africanized bees over stingless bees and other pollinators’ diversity and resilience [26, 28, 30, 32, 73]. Taking into account the aggressive behavior and the relative ecological advantage of the Africanized honey bee over stingless bees, research is important to evaluate at which extent the spread of the Africanized honey bee, including anthropic interference through migratory apiculture, may affect the diversity of native bees. Scientists also sought to assess the impact of the honey bee in plant reproduction, since sometimes this bee acts as pollen “robber”, taking away more pollen than the specific pollinator does and thus compromising the successful reproduction of the plant, with consequent diminished fruit and seed production [29, 30]. This competition does not happen only between *A. mellifera* and other bees, but is a common feature in insect relationships and among the stingless bees [28].

Studies carried out in Brazil and in other countries have shown that the Africanized honey bee, in competition with native bee species, has compromised and/or limited: a) the pollination of native plant species (which many times have pollinator specificity); b) resource availability (pollen, nectar, resin) for native bees; and c) the capacity of native pollinators to get resources by deterrence or expulsion through competition with the exotic bee [25, 27, 29, 31]. Roubik et al. [26] present a classical study on food competition between the exotic and the native bees in Panama. They found out that pollen and nectar harvested by the honey bees were 10–200 times that procured by 17 stingless bee colonies. They did calculations based upon colony populations, food stores and flight range, showing that if Africanized Honey bees persist at a density of one colony per km^2^, colonies of some stingless bee species may disappear after 10 years.

Kremen et al. [74] found no evidence of native bee abundance and diversity decline in response to increased honey bee abundance. They found that the diversity of native bee communities is important in providing crop pollination services because of temporal fluctuations in bee populations, which are highly variable across space and time. According to their findings, a diverse set of species (approximately 20 species) was necessary for sufficient pollination function in one year. Relatively unimportant species in one year became crucial dominants in the next year. They conclude that managing for bee diversity could therefore meet the pollination requirements of a greater number of crops, provide insurance in the event of shortages of any specific pollinator, and options for new or alternative crops, either as a supplement or alternative to current protocols for single-species management.

Regarding the availability of flowers, Kawaiwete beekeepers at Kwaruja village believe that currently there is enough food for all bees. However, teacher Sirawan pointed out that in the future, if the Africanized honey bee population increases, it can lead to greater competition for flowers and nesting places. The beekeepers explained that the flower production is variable and that some flowers are more visited by the bees. In some years, the flower production is high while in others, it is low. Also, for some plants, the flowering period is short and for others it lasts longer. This is consistent with scientific findings on diversity of bees and flowers and the necessity to conserve biodiversity in order to keep pollination sustainable [74].

Dr. Nogueira Neto, Waldemar Ribas Monteiro and Mário Otsuka are critical of the migratory apiculture, as an anthropogenic activity that causes impact on the diversity of native pollinators and on fruit production by plants with specificity for certain pollinators. In this type of apiculture, beekeepers take hundreds of beehives, placing them in a relatively small area, thus the concentration of bees is so high that probably the native bees end up without food. According to Nogueira Neto, migratory apiculture must be strongly controlled, and in Xingu Park it should not be developed.

Dr. Nogueira Neto suggested that in Xingu Park, apiculture is a type of artisanal activity that, if controlled, won’t cause more environmental impact than that resulting from the natural presence of the exotic bee there. APACAME’s representatives emphasized that in Xingu Park, indigenous beekeepers take the colonies and families that already live in nature and bring them to the beehives close to the villages. They do not divide swarms; thus the number of bees is not increased by the activity. There are dozens of beehives in the villages, with a maximum of 25–30 hives, which is small and considered “artisanal” compared to the professional non indigenous beekeepers. Furthermore, they mentioned that in an area of almost 3 million of hectares, there should be food enough (flowers, pollen and nectar) for everyone.

According to Freitas et al. [35], the main factors that can affect bee diversity in Amazon are competition between bees, deforestation and land conversion, and uncontrolled extraction of both *A. mellifera* and stingless bees. Dr. Nogueira Neto observed that when a forest fragment is converted in pasture, the biodiversity is tremendously affected. Grass species are used only eventually for the bees, thus food offer is severely restricted, which can cause local extinction of native insects.

While the *A. mellifera* based business and the struggle to cope with the commercial pollination crises go on, deforestation and climate change, leading to biodiversity declines, has compromised the resilience of ecosystems, and hundreds of insect species might go extinct every day in the Amazon, without being even registered by Western science [75, 76]. In Brazilian Amazon, indigenous lands and other natural protected areas congregate “islands” of diversity, with increasing fragmentation of habitats due to the conversion of natural vegetation in pastures or agricultural fields around these special areas. Amazonian indigenous lands play a fundamental role in the maintenance of biodiversity and in climate balance [77].

APACAME practitioners do not see a conflict in the development of apiculture in Xingu, because they think there is enough resources and area for all species. It is different from migratory or fixed apiculture, they say, practiced in small patches of forest in the Southwest and South of Brazil, for example. This is not the case in Xingu, where conservation procedures are being implemented for apiculture development. The colonies are not being multiplied. This means that indigenous beekeepers are not increasing the number of bees - they are just taking the bees from the forest and keeping them near the village. There is no introduction of swarms from outside the Park. Furthermore, the villages are far from each other, sometimes dozens of kilometers, avoiding overlapping foraging areas for both *Apis* and native bees.

Indigenous beekeepers and managers of natural resources suggested that research on the availability of flowers and on the competition between the exotic *Apis* and the stingless bees should be carried out. They also stated the importance of keeping both *A. mellifera* and stingless bees, and of development of both apiculture and meliponiculture in Xingu:-*I want to try both, together in the same apiary, in a short distance. Then, I have to check out if they are living well or not, but for me the “europa” is not importuning the native bees*”, says Tawareró Ikpeng, beekeeper. Korotowi, a Ikpeng teacher and community leader in Moygu village adds: “- *If we want to increase beehive boxes for Apis and also have native bees in boxes, we cannot anticipate the results of what is going to happen, that the Apis will harm the other bees, but with the experience, watching and doing, we will be able to observe the changes. Nobody knows what happens now, maybe later, as time passes.*”

Fernando de Oliveira, professional beekeeper, suggested that in Xingu the local communities could develop a system for monitoring apiculture and meliponiculture directed towards evaluating competition, flower availability and specificity in pollination or flower visiting. He also thinks that some estimates of capacity of support of the areas around apiaries and meliponaries could be done, as well as calculating minimum distances between them.

Dr. Vera Imperatriz-Fonseca and Dr. Astrid Kleinert suggested further directions for the development of collaborative research between indigenous peoples and researchers, in order to promote both conservation and sustainable use of stingless bees in Brazil [39, 73]. They highlighted that there are few studies done on indigenous peoples and bees in Brazil, and thus we need to concentrate efforts to advance in crucial issues related to “forest sustainability” such as:

- Controlling apiculture in indigenous and other protected areas, to avoid competition with other native bees;
- Stimulating keeping of stingless bees, and the development of techniques for multiplication of beehives;
- Developing research on stingless bee species that visit crop plants and trees which fruits are appreciated by indigenous peoples, stimulating beekeeping for these species;
- Deepening and strengthening the relationship between researchers and indigenous peoples, preserving the rights of both in the dissemination of knowledge;
- Defining priorities for the information to be investigated and setting up protocols for obtaining data.

Dr. Nogueira Neto thinks that if indigenous peoples continue to maintain the natural vegetation and practice both controlled apiculture and keeping of stingless bees, they will benefit from honey production and conservation of native bees’ diversity. Furthermore, they will be giving a great contribution to science through their knowledge of stingless bees, which can be applied in other places for environmental conservation and research on what he calls “forgotten pollinators” [34, 38, 41].

Korotowï Ikpeng, teacher at Moygu village, agrees that if the forest is maintained, there is no risk of extinction for the stingless bees. He emphasizes the need to avoid forest burning, which is a big threat to environmental conservation in Xingu Park.

### Reflection: opportunities and constraints for engaging indigenous and academic knowledge for bee management in the Amazon

The Bees Ecology project contributed to the development and evaluation of approaches for transdisciplinary engagement of indigenous and scientific knowledge for environmental management. Reflecting on the process of engagement, we found both opportunities and constraints or pitfalls, which can inform future initiatives or projects in the Amazon and elsewhere. As a first reflection point, the project was not designed having in mind a reflection on the process of engaging these knowledge systems, hampering potential re-orientation of activities and/or deeper learning by the team of practitioners, experts and indigenous communities. Rather, the project focused on synthesizing perspectives and presenting a response to the problem raised by the funding agency.

Based on the framework by Raymond et al. [6], we consider that the project enabled an initial transdisciplinary exercise of identifying, evaluating and engaging academic and societal perspectives addressing social-environmental problems. The variety of methods utilized, the previous experience and trust existing between the practitioners and indigenous communities, as well as the familiarity of project researchers with academic experts and technicians who participated in the project, were instrumental for the achievement of outcomes in a relatively short period of time, given the limited amount of funds and resources.

Raymond et al. [6] state that the early stages of any inter- or trans-disciplinary project or initiative are critical to create a common ground informing future dialogue and crafting integrative solutions for problems that are user-inspired and user-useful. We consider that, if we had more time and resources, an improved approach to knowledge identification and evaluation would had included an in-depth identification of academic experts working with this theme in the Amazon. After this mapping of experts, they would be interviewed on-line and if possible on-site, and some would be invited to participate in selected project activities on-site, with indigenous communities. For the identification and evaluation of indigenous knowledge, it would have been desirable to conduct semi-structured interviews with a significant proportion of residents in more villages, enabling us to capture the diversity of experiences and perspectives on the theme, and preparing or the workshops, field-excursions and other activities.

We consider that the participatory approach and the use of storytelling during the school workshops were strengths of this project, along with the on-site experiences proportioned by the field walks and mapping activities. Starting with indigenous stories and myths was important to motivate and engage the participants, and to provide a safe and familiar space to the further discussions on the competition between the Africanized honey bee and the stingless bees. However, it was not possible to bring academic experts and technicians during the community workshops and field activities, due to time and financial constraints, in addition to policy regulations regarding the entrance of researchers in Brazilian indigenous lands.

Initiatives for engaging different knowledge systems might thus face epistemological, methodological, political, ethical and practical obstacles [6, 16, 17, 78]. Evely et al. [16] suggest that participants need to be aware of their own and others philosophical and epistemological positions. We believe that, within academic knowledge, disciplinary fields in the social sciences can work as epistemological bridges between diverse knowledge systems. In this case, cultural anthropology could work as a bridging field, providing interpretations of indigenous categories and ways of knowing to biophysical scientists who have no familiarity with these systems, as well as helping to translate or develop understanding of complex concepts from the biophysical sciences to indigenous communities. Thus, it would be important to form an interdisciplinary team of practitioners and researchers to be involved in the design and implementation of project activities, representing relevant disciplinary fields or experiences and improving the process of knowledge identification, engagement and application. Furthermore, leaders in the team, and among indigenous communities and academic experts, would work together to build a shared vision for the project, based on the principle of epistemological pluralism [79]. This philosophical principle recognizes that there may be multiple valid and valuable ways of knowing, employing a continuous process of negotiation between researchers and stakeholders [6, 79].

Based on our experience, we summarize barriers and constraints to indigenous and academic knowledge engagement into five broad categories or themes:

- Ontological perspectives – these refer to definitions of classes and entities by diverse knowledge holders. For instance, this includes systems of classification of bees and concepts such as biodiversity and pollination. Whereas some categories and entities might be comparable between indigenous and academic knowledge, there are disconnects, gaps and conflict between classification and conceptualization of ecological processes and phenomena (e.g. the concept of payment for ecosystem services developed by western science, which conflicts with indigenous peoples’ worldviews and spiritual connections with the environment [3, 80]. A better understanding of nomenclature and linguistics would be instrumental to enable in depth studies of classification, use and management of biodiversity by indigenous peoples.
- Epistemological disconnects and conflicts – epistemology might be understood as a broad theory of knowledge, concerned with how knowledge is acquired, transmitted and validated by human societies [79]. In indigenous societies, the spiritual and the material world are intertwined and knowledge is produced by social activity based on historical and contextual experience, transmitted orally and/or by direct experience, and socially validated. In indigenous Amazonian cosmology, humans and bees have the same status and respect for each other, reflecting a worldview in which animals and plants spiritual and material forms are transitory and dynamic [81]. As the Kĩsêdjê myth says, “there was a time when animals were half humans and humans were half animals.”
- Methodological barriers and opportunities – There are two aspects related to methodological design in transdisciplinary projects. One refers to methods devised to engage participants in the process of knowledge production, and the second is related to methods developed to collect, analyze and present data. Different academic disciplines utilize diverse methods to collect, analyze, organize and present results of research or scholarly inquiry. In inter- or trans-disciplinary initiatives devoted to integrate and generate new knowledge, methods and approaches to a given problem or theme, we suggest that a mixed method approach is desirable to attend to the epistemological specificities of each disciplinary field or knowledge system involved. In the Bees Ecology project and follow-up initiatives, a combination of quantitative, qualitative and participatory methods – such as storytelling, participatory mapping, ethnographic and biophysical (mapping, population inventories, inventories of melliferous plants, etc.) methods - would had provided a more comprehensive picture of the problem under analysis, also allowing for knowledge exchange and integration towards context relevant management actions.
- Policy and power opportunities and constraints – there are multiple political and power barriers and constraints academic and indigenous knowledge engagement. Describing and analyzing these in detail is beyond the scope of this article. We thus focus on the topics of power and colonialism and intellectual property rights, which we believe are relevant to inform a reflection of initiatives for transdisciplinary co-production of knowledge informing environmental management. From a power perspective, indigenous knowledge systems might be wrongly appropriated and communicated by academic scholars lacking a critical perspective of the position and limits of Western science to present and represent such complex knowledge systems [78, 82]. Postcolonial authors have questioned the assumption that science is a politically neutral enterprise in search of an ultimate truth [83–85]. According to Seth [84], Western science has been historically used as a tool to support ideological and economic interests of powerful nations and elites. Thus, in any transdisciplinary initiative aiming to engage diverse knowledge systems, one needs to take into account issues of power within academic fields and between these and non-academic systems, such as IK. As a starting point, it is important that participants recognize that all knowledge systems are equally important and valid to solve the problem in hand, and adopt “epistemological pluralism” as a working concept [79]. Legal frameworks might enable the protection of indigenous intellectual property rights, but at the same time can hamper efforts to study, manage and conserve biodiversity among indigenous peoples. In a follow-up initiative based on this study, it would be desirable to make provisions to enable the scientific identification for the ethnospecies of stingless bees identified by indigenous communities. This would provide an opportunity for knowledge exchange across indigenous peoples involved in the project, and, more importantly, it would allow for identification of indicator species, key interactions and research and conservation gaps and priorities. At the same time, necessary precautionary actions should be taken to protect indigenous communities and their knowledge of biodiversity against violation of rights and improper use for commercial purposes.
- Funding constraints and lack or inconsistent follow-up – research is always guided by a balance between methodological rigor and availability of time and funds. Time and funding constraints may limit the potential reach and deeper engagement and integration of knowledge within and between indigenous communities as well as between them and academic experts, practitioners and policy-makers. The lack of follow-up and adaptive learning among NGO practitioners, technicians and academics is also a critical problem that affects the application of transdisciplinary knowledge. Reflection on processes and projects might be a platform to encourage adaptive learning and continuity in participatory projects developed with indigenous communities, even if they focus on different problems.

## Conclusion

Indigenous peoples from Xingu Park participating in this project have incorporated the Africanized honey bee into their knowledge systems and cosmovision in a rather short period of time. Interestingly, the introduced species was incorporated into myths and stories along with existing species. This has implications for the field of human ecology and other related scientific areas, in the recognition of the dynamic and mobile aspects of indigenous knowledge in contemporary contexts.

The Africanized honey bee was compared by indigenous participants to the non-indigenous western colonizers, who came from far to colonize Amazonian landscapes, with which indigenous societies have had to learn to adapt to and coexist with. Advantage or prejudice is not a straight forward question to answer, since the Africanized honey bee brought both benefits and problems to stingless bee communities, who can be compared to the different indigenous ethnic groups that historically inhabit and manage Amazonian landscapes.

The introduction of exotic animal and plant species into continents and environmental conditions is not a new problem, but an action that has been practiced by governments, scientists and people for millennia. However, often the environmental and social impacts that these exotic species can cause are not sufficiently assessed before the process is done, and the effects may not be felt in a short length of time. Thus, release of exotic or hybrid species in the wild should be critically controlled by government, researchers and managers, and should not be done without consultation and discussion with relevant societal actors, such as indigenous peoples.

Local knowledge is a starting point for participatory construction of knowledge or for engaging different knowledges to solve a specific problem. As Whyte [86] suggests, traditional ecological knowledge might be approached as a collaborative concept, serving to invite diverse populations to continually learn from one another about how each approaches the question of knowledge in the first place, and how these different approaches might be engaged to better steward natural resources for enhanced conservation and human well-being. It is important to recognize that there are multiple barriers and constraints to transdisciplinary engagement between academic, technical, and indigenous knowledge systems. These include epistemological, political, and financial constraints.

Both academic and indigenous persons involved in this study recognize that diversity is a key aspect in keeping both exotic and native species in balance and thus avoiding heightened competition and extinction. It is important to recognize the role of indigenous peoples’ knowledge systems in creating, enhancing and conserving biodiversity in both local and landscape scales. Keeping diversity in indigenous lands may also entail developing economic alternatives compatible with traditional lifestyles. Artisanal apiculture and meliponiculture may be practiced as sustainable activities that potentially contribute to the maintenance of biological and cultural diversity in Amazonian landscapes.

## Footnotes

1 Instituto Socioambiental (ISA): www.socioambiental.org

2 APACAME: http://www.apacame.org.br/
